# Association between circulating inflammatory proteins and gout: A Mendelian randomization study

**DOI:** 10.1097/MD.0000000000042379

**Published:** 2025-05-16

**Authors:** Xiaochao Xie, Yanjie Song, Wenwen Chen, Hui Zhao, Nan Chu, Fang Wang

**Affiliations:** a Department of Endocrinology and Metabolism, The Affiliated Hospital of Qingdao University, Qingdao, People’s Republic of China; b Department of Endocrinology, Qingdao Hiser Hospital Affiliated of Qingdao University (Qingdao Traditional Chinese Medicine Hospital), Qingdao, People’s Republic of China; c Department of Cardiology, Qingdao Hiser Hospital Affiliated of Qingdao University (Qingdao Traditional Chinese Medicine Hospital), Qingdao, People’s Republic of China.

**Keywords:** bidirectional, circulating inflammatory proteins, gout, Mendelian randomization, meta-analysis

## Abstract

Clinical studies have consistently demonstrated that inflammation is a critical factor in the pathophysiology and progression of gout. This study aims to explore the causal relationship between CIPs and gout, utilizing MR in conjunction with meta-analyses. We utilized genetic data pertaining to gout from the GWAS which involved 3576 cases and 147,221 control participants. A total of 132 CIPs were extracted from the GWAS data to identify SNPs associated with gout. The primary analytical approach was the IVW method. Sensitivity analyses indicated no pleiotropy or heterogeneity. The IVW results revealed that several CIPs were associated with gout in European populations. The analysis results indicate that FGF-21, MMP-1, G-CSF, and IFN-γ are involved in the pathogenesis of gout, and gout may influence the expression of CXCL1, IL-1Ra, and TNF-α. Consequently, targeted research focusing on specific CIPs could provide a promising strategy for the treatment and prevention of gout, offering potential therapeutic targets for the underlying inflammatory mechanisms of the disease.

## 1. Introduction

Gout is a disorder characterized by inflammation of the joints due to elevated levels of uric acid in the body. This disorder results from dysregulation in purine metabolism, leading to the accumulation of monosodium urate (MSU) crystals within and around the articular structures. The deposition of these crystals triggers an inflammatory response, ultimately resulting in gouty arthritis.^[1]^ Recent evidence has increasingly highlighted the central role of chronic inflammation in the pathogenesis of gout. Over the past 2 decades, significant progress has been made in understanding the innate immune mechanisms that drive gouty inflammation. The gold standard for diagnosing gout remains the identification of MSU crystals in gouty tophi via microscopy.^[2]^

It has been well established that the deposition of MSU crystals in the joints triggers the activation of immune cells, including macrophages, neutrophils, and monocytes. These cells release a range of circulating inflammatory proteins (CIPs), such as tumor necrosis factor-alpha (TNF-α), interleukin-1β (IL-1β), interleukin-6 (IL-6), and interleukin-18 (IL-18), which initiate and amplify the inflammatory cascade that underpins the pathogenesis of gout.^[3,4]^ Among these, the interleukin-1(IL-1) family of cytokines is particularly critical, playing a pivotal role in both innate immunity and the regulation of inflammatory processes. IL-1 family members mediate immune responses through diverse mechanisms, including the processing and secretion of precursor proteins, modulation of gene expression, and interaction with specific receptors.^[5]^ The IL-1 family consists of 13 members, including IL-1β, IL-6, and IL-18, all of which are involved in the inflammatory response associated with gout. Furthermore, TNF-α is another key pro-inflammatory cytokine that contributes significantly to the exacerbation of gout attacks. Collectively, these inflammatory mediators intensify both the inflammatory response and the pain associated with gout through complex signaling pathways and cell-to-cell interactions. A deeper understanding of the mechanisms by which these inflammatory factors exert their effects is crucial for the development of novel therapeutic strategies, such as the use of IL-1β antagonists (e.g., anakinra) to mitigate the symptoms of gout.^[6]^ However, research on the role of other CIPs in gout remains limited. Moreover, establishing causal relationships between specific cytokines and gout is challenging, owing to the confounding factors and reverse causality inherent in clinical and observational studies.^[7]^

Mendelian Randomization (MR) is a research methodology that leverages genetic variation as instrumental variables (IVs) to assess causal relationships between risk factors and specific diseases.^[8]^ In MR analysis, genetic variation adheres to the principle of random allele distribution during meiosis, akin to the randomization process in controlled trials. Consequently, this approach mitigates several limitations inherent in observational studies, such as confounding, reverse causality, and selection bias, while also circumventing the logistical constraints of randomized controlled trials. Previous studies have demonstrated that genetic variation in individuals can influence the incidence, progression, and clinical outcomes of gout, with identified genetic polymorphisms potentially modulating both the risk and severity of the disease. To date, MR has not been applied to explore the causal relationship between CIPs and gout.

In this study, we employ two-sample MR analysis to investigate the causal relationships between multiple CIPs and the development of gout, and integrate data from Genome-wide Association Study (GWAS) with MR analysis to investigate the causal associations between various CIPs and the risk of gout. The aim of this study is to clarify the role of CIPs in gout pathogenesis and to establish a theoretical framework for future research on potential pharmacological targets for gout, which is of considerable clinical significance.

## 2. Materials and methods

### 2.1. Ethics statement

Our study involved human participants’ data which are from the IEU open GWAS project and the EBI GWAS Catalog project (obtained date: October 07, 2024). The data from these 2 projects are publicly available. Ethical approval was not necessary for our study.

### 2.2. Study design

**Figure 1.**
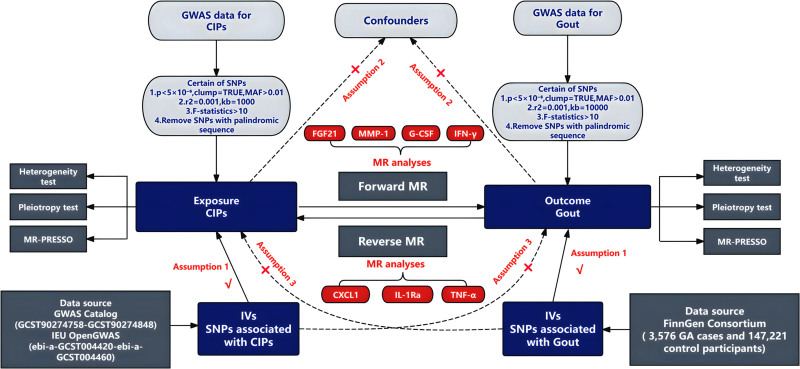
Bidirectional MR research flowchart for CIPs and gout. This figure elucidates the 3 foundational assumptions of MR: (1) Relevance: SNPs robustly associated with exposure, independence, and exclusion restriction; (2) Independence: SNPs not associated with confounders; (3) Exclusion restriction: SNPs only associated with outcome through exposure. CIPs = circulating inflammatory proteins, IVs = instrumental variables, SNPs = single nucleotide polymorphisms, MR = Mendelian randomization.

MR is based on 3 fundamental assumptions: relevance: the IVs used in the study must be strongly associated with the exposure factor; independence: the IVs must be independent of any confounding factors that could influence the exposure-outcome relationship; and exclusion restriction: the exposure factor should be the sole pathway through which the IVs affect the outcome.^[9]^ To comprehensively identify CIPs associated with gout, this study conducted an extensive screening of GWAS data, resulting in a dataset comprising 41 previously published CIPs and 91 newly identified CIPs.^[10]^To further explore the causal relationship between CIPs and gout, we performed a bidirectional MR analysis. In the forward MR analysis, CIPs were treated as exposure factors, with gout as the outcome variable, to assess the causal link between CIPs and gout. In the reverse MR analysis, gout was considered as the exposure factor and CIPs as the outcome variable, enabling the investigation of the potential causal influence of gout on CIPs, as illustrated in Figure 1.

### 2.3. Data source

The GWAS data for 91 CIPs were obtained from 14,824 participants of European ancestry across 11 independent cohorts, and a genome-wide protein quantitative trait loci (pQTL) analysis was performed for these 91 CIPs.^[11]^ These data are available through the EBI GWAS Catalog project (IDs ranging from GCST90274758 to GCST90274848).

The GWAS data for 41 inflammatory factors were derived from combined analyses of 3 independent cohorts, comprising a total of 8293 Finnish participants.^[12]^ These data can be accessed via the MRC IEU open GWAS project (IDs ebi-a-GCST004420 to ebi-a-GCST004460).

Single nucleotide polymorphisms (SNPs) associated with gout were extracted from the FinnGen database, which includes 3576 European cases and 147,221 controls. This dataset is also available for download from the IEU open GWAS project (ID finn-b-M13_GOUT). Since the data used in this study are publicly available from GWAS databases, ethical approval and informed consent were not required.

### 2.4. Selection of IVs

To satisfy the first MR assumption regarding the strong association between SNPs and cytokines, it is crucial to select SNPs that are significantly associated with CIPs at the genome-wide level (*P* < 5 × 10⁻⁸). However, it is important to acknowledge the limitations of using this stringent threshold for SNP selection in certain human phenotypes. In the context of this bidirectional MR analysis, we encountered such limitations when applying the *P* < 5 × 10⁻⁸ threshold. To obtain more robust IVs, we relaxed the genome-wide significance threshold and set the SNP inclusion criterion to *P* < 5 × 10⁻⁶.^[13]^ Relaxation of the genome-wide significance threshold followed previously validated approaches for MR analyses with limited instrument strength.^[13–20]^ To minimize the influence of linkage disequilibrium (LD) on our results, we implemented a two-stage LD pruning and clumping procedure based on established methodologies.^[7,14,21–24]^ First, genome-wide LD pruning was performed using PLINK v1.9 “indep-pairwise” function^[21]^ with a 10,000 kb sliding window and stringent *r*² threshold of 0.001. This approach, recommended by Purcell et al for capturing long-range LD regions,^[21]^ effectively removed redundant SNPs within the exposure dataset.

The selected parameters (10,000 kb window; *r*^2^ < 0.001) were validated by Burgess et al as optimal thresholds for ensuring SNP independence in Mendelian randomization studies.^[7,23]^ To estimate the proportion of variance explained by each SNP for the exposure variable (i.e., the *R*^2^ value), the following formulas were applied:


$$\begin{array}{lll} & R^{2}=\frac{2\times EAF\times \left(1-EAF\right)\times beta^{2}}{\left(2\times EAF\times \left(1-EAF\right)\times beta^{2}\right)+\left(2\times EAF\times \left(1-EAF\right)\times N\times SE{\left(beta\right)}^{2}\right)};\; & F=\frac{R^{2}\left(N-2\right)}{1-R^{2}} \end{array}$$

The two formulas provided involve the effect allele frequency (EAF), where 1 − EAF represents the frequency of the non-effect allele, N denotes the sample size for the exposure variable, beta refers to the estimated genetic effect, and SE represents the standard error of the genetic effect. These formulas quantify the degree to which each SNP accounts for the variability in the exposure variable, with a higher *R*^2^ value indicating a stronger explanatory power of the SNP for the exposure. In MR, selecting SNPs with higher *R*^2^ values as instrumental variables typically enhances both the statistical power and the reliability of the analysis. To assess the strength of genetic instrumental variables in MR, a higher *F*-statistic suggests a stronger SNP instrument, with a threshold value of 10 or greater considered indicative of a robust instrument.^[25]^

To further ensure the validity of IVs, we performed rigorous quality control steps to exclude pleiotropic and weak instruments: Pleiotropy removal: SNPs showing significant associations (*P *< 1 × 10⁻⁵) with potential confounders were identified via PhenoScanner V2 and excluded. Horizontal pleiotropy was assessed using MR-Egger intercept test (*P *> .05) and MR-PRESSO global test (*P *> .05). Outlier SNPs detected by MR-PRESSO were iteratively removed. Weak instrument filtering: All retained SNPs had *F* statistics > 10. SNPs with *F* < 10 were excluded. Heterogeneity checks: Cochran’s Q test indicated no significant heterogeneity among SNPs. Sensitivity analyses using leave-one-out method confirmed no single SNP drove the causal effect.

In this study, we assume that the selected genetic instruments satisfy the independence assumption, meaning that these instruments are not associated with any known or unknown confounders in the study. In other words, the variation in the instruments affects the outcome variable only through the exposure variable, and not through other potential confounding factors. To validate this assumption, we conducted a series of sensitivity analyses and ruled out factors that may affect the independence of the instruments.

### 2.5. Statistical analysis

This study employed bidirectional MR to assess causal relationships, utilizing the inverse variance weighted (IVW) method, which was widely regarded as the most effective approach in MR due to its high statistical power. Supplementary analyses were performed using the MR Egger, Weighted Median, Simple Mode, and Weighted Mode methods.^[26]^ The odds ratio (OR) and its corresponding 95% CI derived from the bidirectional MR analysis were used to evaluate the causal effect of the exposure on the outcome. Various visualization tools, including forest plots, scatter plots, and funnel plots, were employed to present the results. Additionally, a circular heatmap was generated by combining *P* values from the 5 MR methods with the OR from the IVW method.

To evaluate the stability and reliability of the MR results, this study conducted multiple sensitivity analyses employing various methods. MR-Egger was used to assess and adjust for the potential influence of horizontal pleiotropy. A significant non-zero intercept in MR-Egger suggests the presence of interference from other factors on the effect of the instrumental variable on the outcome. By estimating the intercept, more reliable causal estimates can be obtained in the presence of pleiotropy, thereby reducing bias induced by pleiotropy.^[27]^ MR-PRESSO evaluates residuals and outliers of the instrumental variables to identify potential pleiotropic effects. If abnormal instrumental variables are identified, removing these outliers and applying corrections ensures more robust results.^[28]^ Additionally, a leave-one-out analysis was performed to assess the potential influence of individual SNPs on the MR estimates, thereby enhancing the reliability and stability of the results.^[29]^ To assess heterogeneity, Cochran’s Q tests were conducted for both the IVW and MR-Egger methods. A *P* value below .05 indicates the presence of heterogeneity, which is assessed using a random-effects model, while a *P* value of .05 or greater is evaluated with a fixed-effects model.^[30]^ To reduce the probability of erroneously rejecting the null hypothesis due to multiple testing, Bonferroni correction was applied by dividing the original significance threshold by the number of independent tests. Therefore, a *P* value of less than 3.79 × 10^−4^ (0.05/132) indicates a significant association, whereas *P* values between 3.79 × 10^−4^ and 0.05 suggests a suggestive association.^[31]^ Furthermore, a meta-analysis was conducted on the results from the same CIPs across 2 datasets.^[32]^ Statistical analyses were performed using R software (version 4.4.0), with the following packages: TwoSampleMR, MendelianRandomization, circlize, ComplexHeatmap, tidyverse, ggpubr, and MRPRESSO.

### 2.6. Statistical power calculations

To evaluate the reliability of causal inferences, we performed statistical power calculations for all inflammatory factors included in the bidirectional MR analysis. Following the methodology of Burgess,^[7]^ we estimated power based on the following parameters: Instrument strength: Genetic instruments were restricted to SNPs with *F*-statistics > 10 to minimize weak instrument bias; Effect size range: We assumed OR from 1.1 to 1.5 for the exposure-outcome relationship; and Sample size: The sample sizes of both exposure and outcome GWAS datasets were incorporated into the power formula. Full results of these calculations are provided in Table S7, Supplemental Digital Content, https://links.lww.com/MD/O842 and Figure S7, Supplemental Digital Content, https://links.lww.com/MD/O843.

## 3. Results

The study incorporated a total of 133 GWASs, including 132 for CIPs and one for gout (Table S1, Supplemental Digital Content, https://links.lww.com/MD/O842). The F-statistics for all instrumental variables were greater than 10, demonstrating the robustness of the selected SNPs (Table S2, Supplemental Digital Content, https://links.lww.com/MD/O842).

### 3.1. Causal effects of CIPs on gout

**Figure 2.**
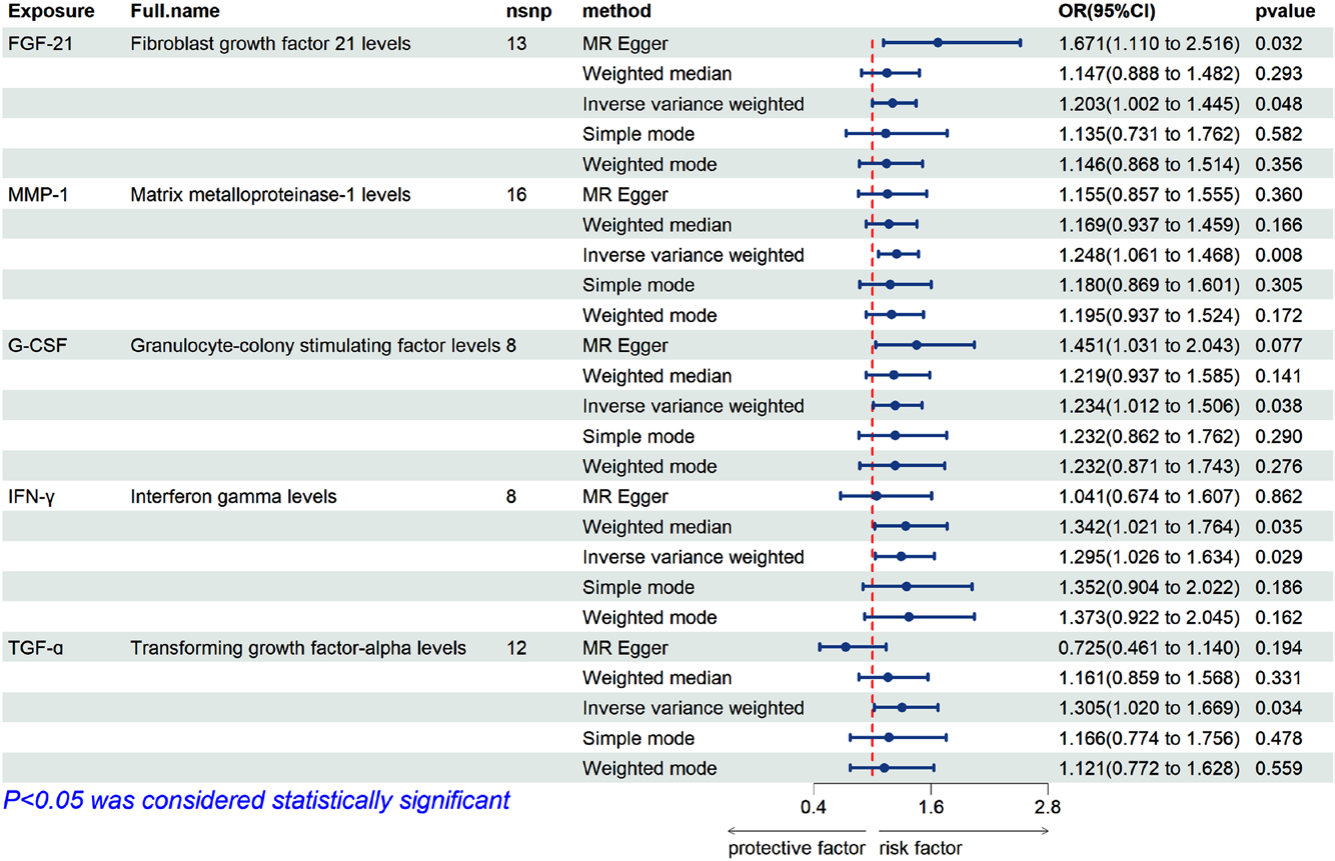
The positive results of the forward MR analysis conducted with the Inverse variance weighted, MR-Egger, Weighted median, Simple mode, and Weighted mode method. FGF21 = Fibroblast Growth Factor 21, MMP-1 = Matrix Metalloproteinases-1, nsnp = the number of single-nucleotide polymorphisms used in the analysis, OR = odds ratio.

**Figure 3.**
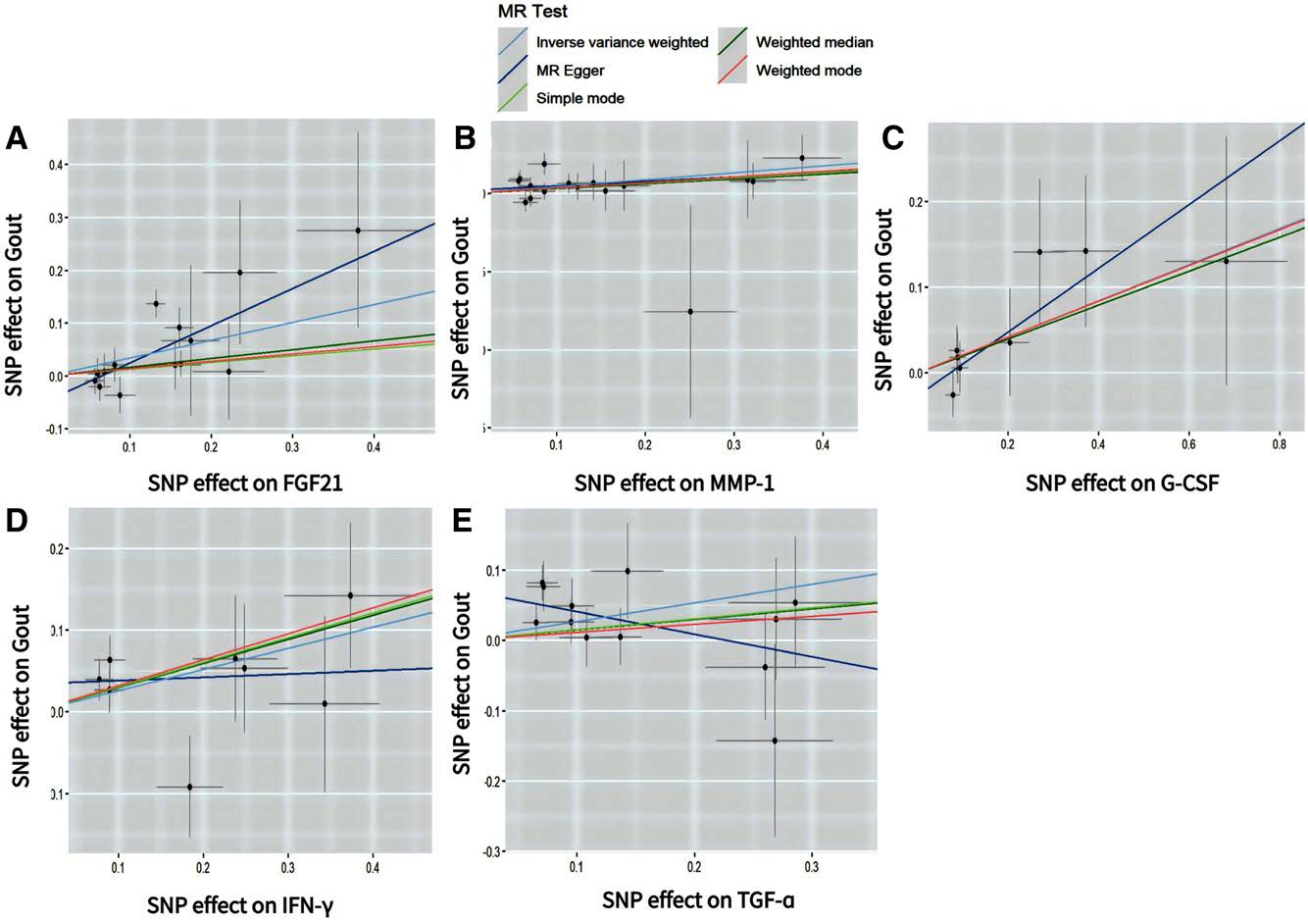
Scatter plots of the causal effects of CIPs associated SNPs on gout. (A) FGF21, Fibroblast Growth Factor 21; (B) MMP-1, Matrix metalloproteinase-1; (C) G-CSF, Granulocyte Colony-Stimulating Factor; (D) IFN-γ, Interferon-gamma; (E) TGF-_α_, Transforming Growth Factor Alpha. CIPs = circulating inflammatory proteins, SNPs = single nucleotide polymorphisms.

**Figure 4.**
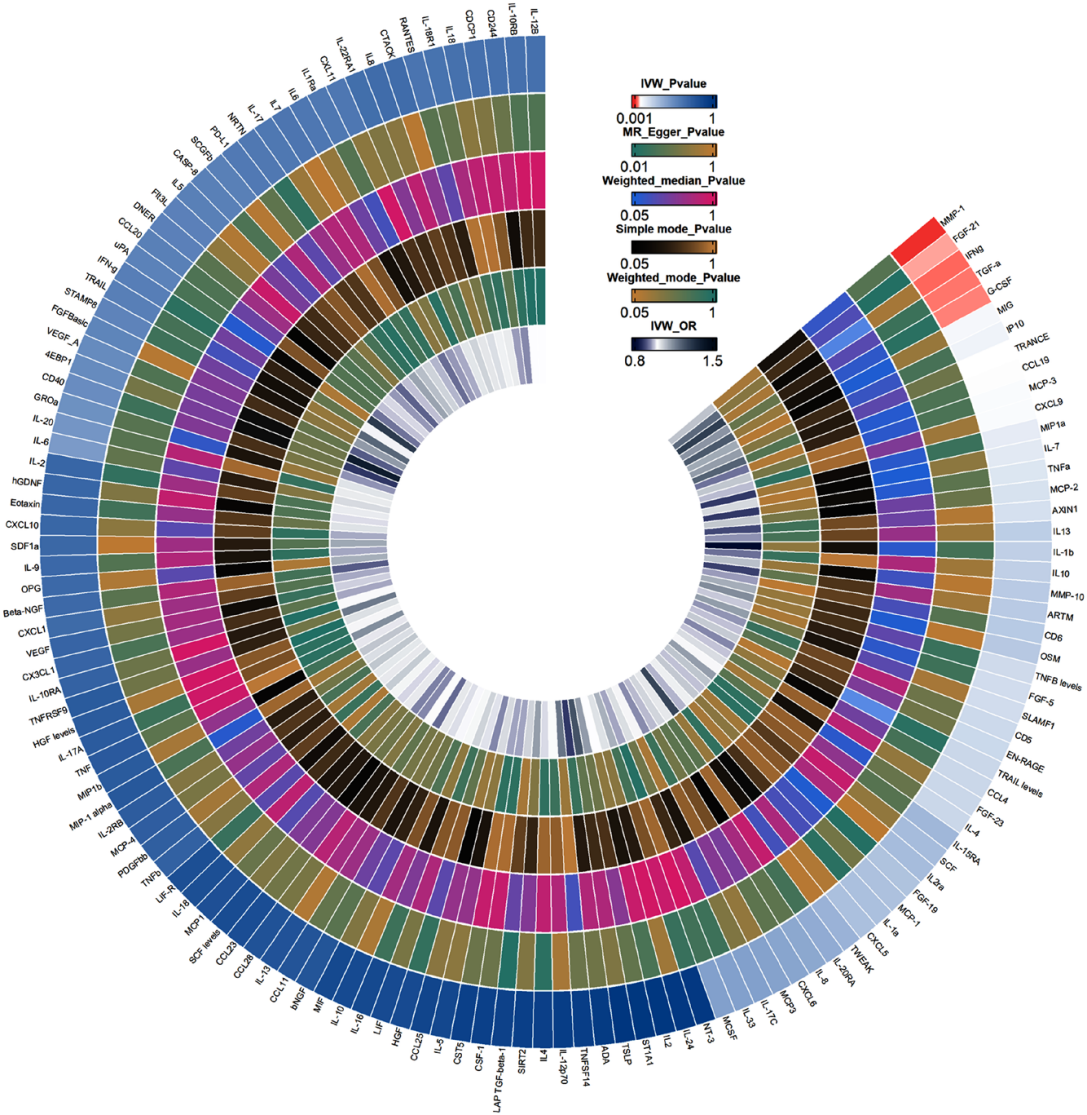
Circular heatmap of forward MR analysis. The circular heatmap, from outer to inner layers, represents the *P* values obtained from 5 MR methods (IVW, MR Egger, Weighted median, Simple mode, Weighted mode), with the innermost layer depicting the OR values obtained from the IVW method. IVW = Inverse variance weighted.

In the IVW analysis of forward MR, 5 CIPs were suggestively and positively associated with gout, including fibroblast growth factor 21 (FGF-21) (OR = 1.203; 95% confidence interval [CI]: 1.002–1.445; *P* = .048), matrix metalloproteinases-1 (MMP-1) (OR = 1.248; 95% CI: 1.061–1.468; *P* = .008), granulocyte colony-stimulating factor (G-CSF) (OR = 1.234; 95% CI: 1.012–1.506; *P* = .038), interferon-gamma (IFN-γ) (OR = 1.295; 95% CI: 1.026–1.634; *P* = .029), and transforming growth factor alpha (TGF-α) (OR = 1.305; 95% CI: 1.020–1.669; *P* = .034) (Fig. 2). The forest plot results from the IVW and MR-Egger analyses for these 5 CIPs are presented in Figure S1, Supplemental Digital Content, https://links.lww.com/MD/O843. Scatter plots suggested a positive correlation between these 5 CIPs and gout (Fig. 3). Bonferroni correction for multiple comparisons indicated that the association between these 5 CIPs and gout was positive (3.79 × 10^−4^ < *P* < .05). The “leave-one-out” analysis confirmed the robustness of the MR findings (Figure S2, Supplemental Digital Content, https://links.lww.com/MD/O843). The forest plot for the 132 CIPs using the IVW method in the forward MR analysis is shown in Figure S3, Supplemental Digital Content, https://links.lww.com/MD/O843, and the heatmap showing the 132 CIPs as exposures and gout as the outcome is presented in Figure 4.The results of the causal relationship between 132 CIPS and gout in the forward MR analysis are presented in Table S3, Supplemental Digital Content, https://links.lww.com/MD/O842.

The MR-Egger intercept analysis revealed no evidence of horizontal pleiotropy for FGF-21, MMP-1, G-CSF, and IFN-γ, with intercepts of FGF-21 (intercept = −0.040, *P* = .106), MMP-1 (intercept = 0.01, *P* = .551), G-CSF (intercept = −0.005, *P* = .964), and IFN-γ (intercept = 0.034, *P* = .291). However, horizontal pleiotropy was observed for TGF-α (intercept = 0.073, *P* = .017). MR-PRESSO results indicated no significant heterogeneity for these 5 CIPs, with FGF-21 (RSSobs = 11.414, *P* = .66), MMP-1 (RSSobs = 13.968, *P* = .685), G-CSF (RSSobs = 5.515, *P* = .784), IFN-γ (RSSobs = 10.58, *P* = .343), and TGF-α (RSSobs = 18.47, *P* = .2) (Table 1). Although the *P* value for TGF-α in MR-PRESSO was greater than .05, indicating no significant instrumental variable outliers or heterogeneity caused by outliers, the MR-Egger intercept analysis detected significant horizontal pleiotropy, suggesting that TGF-α may influence the outcome through pathways unrelated to the exposure. The use of uncorrected effect estimates could lead to bias.^[7]^ Therefore, TGF-α was considered to exhibit significant pleiotropy and was excluded from the forward results of the IVW analysis. All Cochran’s Q test *P* values were greater than .05, indicating no heterogeneity; thus, all analyses were conducted using a fixed-effects model (Table 1).

**Table 1:** Heterogeneity test, pleiotropy test, and MR-PRESSO results for positive CIPs and gout in forward and reverse MR

Exposure	Outcome	Heterogeneity test				Pleiotropy test			MR-PRESSO	
		Method	Q	Q_df	Q_*P* value	Egger intercept	SE	*P* value	Global test RSSobs	Global test *P* value
FGF21	Gout	MR Egger	6.473	11	.84	−0.04	0.023	.106	11.414	.66
		IVW	9.57	12	.654					
MMP-1	Gout	MR Egger	12.432	23	.779	0.01	0.017	.551	13.968	.685
		IVW	12.805	24	.702					
G-CSF	Gout	MR Egger	3.094	20	.116	−0.005	0.09	.964	5.515	.784
		IVW	4.397	21	.088					
IFN-γ	Gout	MR Egger	6.837	6	.336	0.034	0.03	.291	10.58	.343
		IVW	8.365	7	.302					
TGF-α	Gout	MR Egger	7.472	10	.680	0.073	0.026	.017	18.47	.2
		IVW	15.655	11	.154					
Gout	CXCL1	MR Egger	20.175	15	.165	0.023	0.022	.313	24.296	.183
		IVW	21.641	16	.155					
Gout	IL1-Ra	MR Egger	12.693	15	.626	0.04	0.018	.044	19.447	.374
		IVW	17.541	16	.352					
Gout	TNF-α	MR Egger	11.642	15	.706	0.012	0.019	.523	13.508	.762
		IVW	12.07	16	.74					

CXCL1 = CXC motif chemokine ligand 1, FGF21 = Fibroblast Growth Factor 21, G-CSF = Granulocyt Colony-Stimulating, IFN-γ = Interferon-gamma, IL-1Ra = Interleukin 1 Receptor Antagonist, IVW = Inverse variance weighted, MMP-1 = Matrix metalloproteinase-1, MR = Mendelian randomization, TGF-α = Transforming Growth Factor Alpha, TNF-α = Tumor Necrosis Factor-alpha Factor.

### 3.2. Causal effects of gout on CIPs

**Figure 5.**
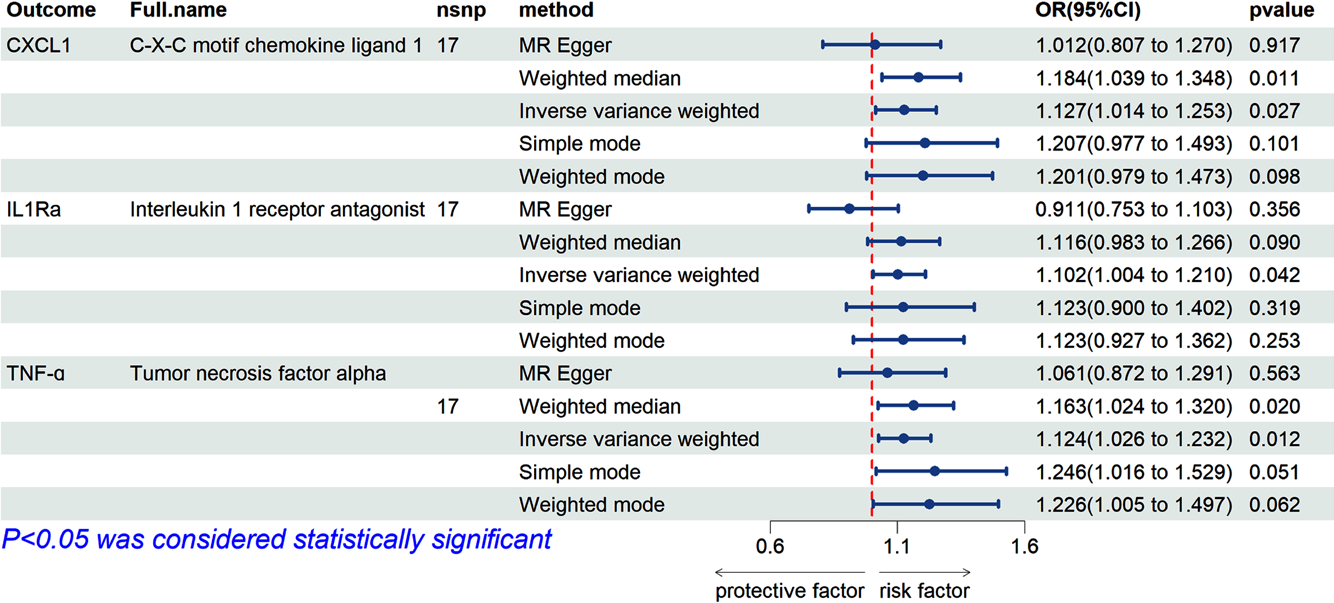
The positive results of the reverse MR analysis conducted with the Inverse variance weighted, MR-Egger, Weighted median, Simple mode, and Weighted mode method. CXCL1 = CXC motif chemokine ligand 1, IL-1Ra = Interleukin 1 Receptor Antagonistm, nsnp = the number of single-nucleotide polymorphisms used in the analysis, OR = odds ratio, TNF-α = Tumor Necrosis Factor-alpha.

**Figure 6.**
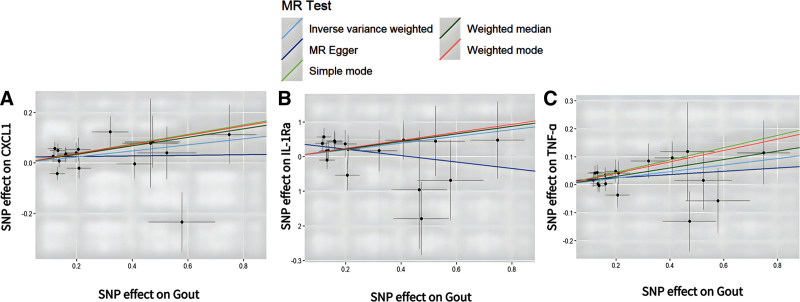
Scatter plots of the causal effects of gout associated SNPs on CIPs. (A) CXCL1, CXC motif chemokine ligand 1; (B) IL-1Ra, Interleukin 1 Receptor Antagonist; (C) TNF-α, Tumor Necrosis Factor-alpha. CIPs = circulating inflammatory proteins, SNPs = single nucleotide polymorphisms.

**Figure 7.**
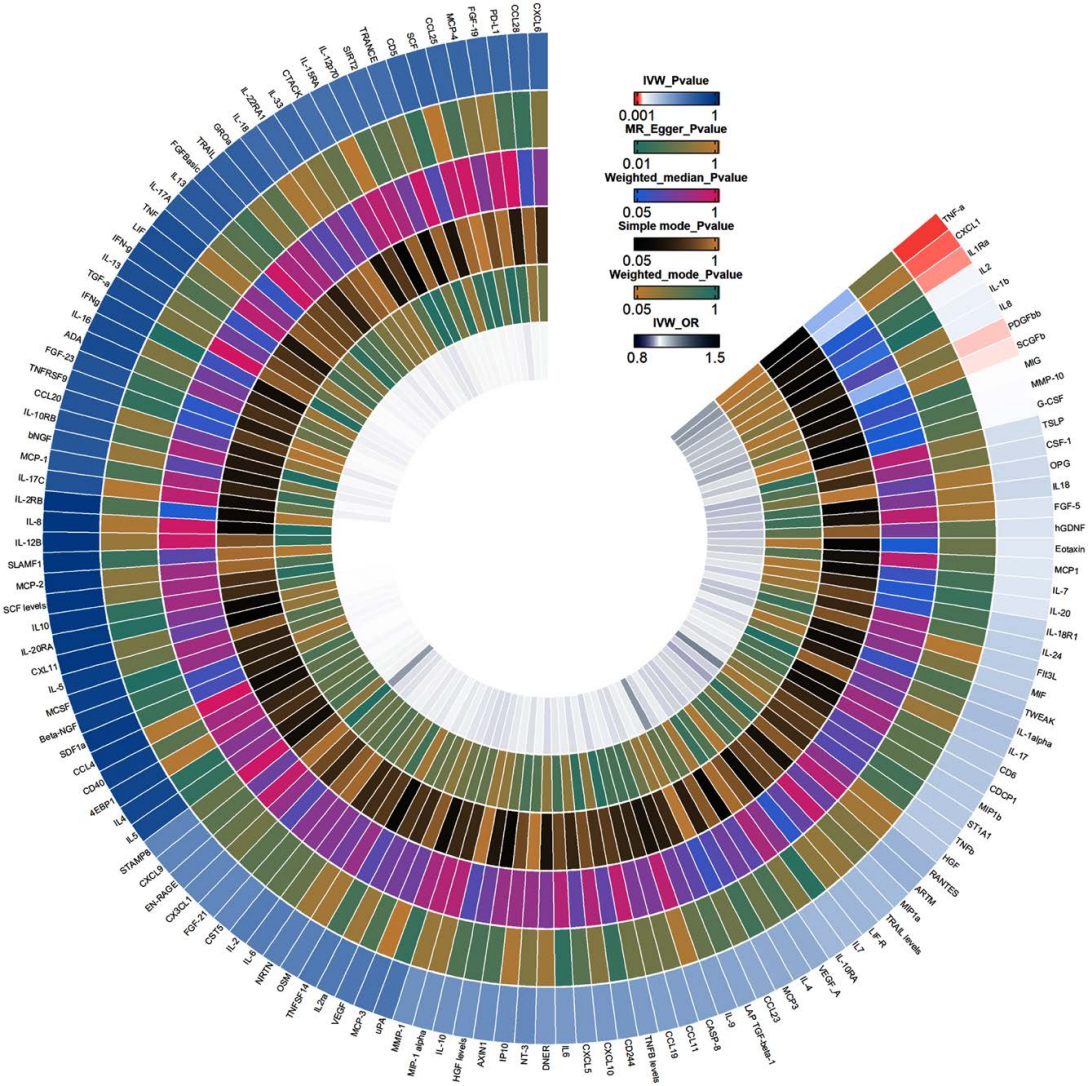
Circular heatmap of reverse MR analysis. The circular heatmap, from outer to inner layers, represents the *P* values obtained from 5 MR methods (IVW, MR Egger, Weighted median, Simple mode, Weighted mode), with the innermost layer depicting the OR values obtained from the IVW method. IVW = Inverse variance weighted.

Reverse MR analysis revealed a suggestive positive association between gout and CXC motif chemokine ligand 1 (CXCL1) (OR = 1.127; 95% CI: 1.014–1.253; *P* = .027), interleukin-1 receptor antagonist (IL-1Ra) (OR = 1.102; 95% CI: 1.004–1.210; *P* = .042), and TNF-α (OR = 1.124; 95% CI: 1.026–1.232; *P* = .012) (Fig. 5). Scatter plots suggested a positive correlation between these 3 CIPs and gout (Fig. 6). After Bonferroni correction for multiple comparisons, a suggestive association between gout and these 3 CIPs was confirmed (3.79 × 10^−4^ < *P* < .05). Figure S4, Supplemental Digital Content, https://links.lww.com/MD/O843 provides the forest plot of IVW and MR-Egger analysis results for these 3 CIPs. The “leave-one-out” analysis confirmed the robustness of the MR findings (Figure S5, Supplemental Digital Content, https://links.lww.com/MD/O843). Additionally, the forest plot for the 132 CIPs in the reverse MR analysis using the IVW method is shown in Figure S6, Supplemental Digital Content, https://links.lww.com/MD/O843. The heatmap with gout as the exposure and CIPs as the outcome is presented in Figure 7. The results of the causal relationship between 132 CIPS and gout in the reverse MR analysis are presented in Table S4, Supplemental Digital Content, https://links.lww.com/MD/O842.

MR-Egger intercept analysis showed no evidence of horizontal pleiotropy for CXCL1 (intercept = 0.023, *P* = .313), IL-1Ra (intercept = 0.04, *P* = .044), or TNF-α (intercept = 0.012, *P* = .523). MR-PRESSO results indicated no significant heterogeneity for these 3 CIPs: CXCL1 (RSSobs = 24.296, *P* = .183), IL-1Ra (RSSobs = 19.447, *P* = .374), and TNF-α (RSSobs = 13.508, *P* = .762). Cochran’s Q test *P* values were all greater than .05, indicating no heterogeneity, and thus, all analyses were conducted using a fixed-effects model (Table 1).

### 3.3. Meta-analysis

**Figure 8.**
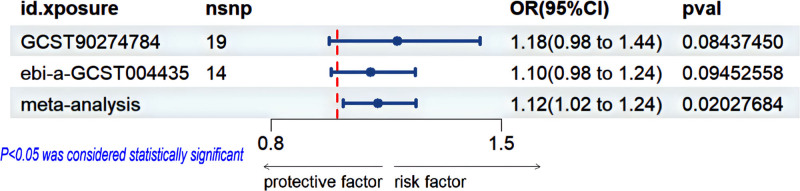
Forest plot of the relationship between gout and CXCL1 based on meta-analysis results from the reverse MR analysis. CXCL1 = CXC motif chemokine ligand 1, MR = Mendelian randomization, nsnp = the number of single-nucleotide polymorphisms used in the analysis, OR = odds ratio.

To strengthen the robustness of the findings, a meta-analysis was conducted for 23 pairs of MR results derived from two distinct data sources: the IEU OpenGWAS and the EBI GWAS Catalog. The meta-analysis revealed that an elevated risk of gout was significantly associated with increased levels of CXC motif chemokine ligand 9 (CXCL9), also known as monokine induced by gamma interferon (MIG), with an OR of 1.12 (95% CI: 1.02–1.24; *P* = .020), based on the IVW method after combining the MR results from both datasets (Fig. 8). No significant correlation was observed between gout and other CIPs. Additionally, heterogeneity and sensitivity analyses revealed no noteworthy associations (Tables S5 and S6, Supplemental Digital Content, https://links.lww.com/MD/O842).

### 3.4. Power analysis of MR analysis

We performed comprehensive statistical power calculations for all CIPs, with detailed results provided in Table S7, Supplemental Digital Content, https://links.lww.com/MD/O842. Among the 7 CIPs demonstrating significant causal associations with gout after Bonferroni correction, all achieved 100% statistical power under the current sample size (Figure S7, Supplemental Digital Content, https://links.lww.com/MD/O843), indicating high robustness in prioritizing these factors as potential therapeutic targets.

For non-significant associations, some CIPs exhibited insufficient statistical power (<80%), likely attributable to limited GWAS sample sizes for the corresponding exposures. Sensitivity analyses employing weighted median and MR-Egger regression revealed consistent directionality of effect estimates, suggesting no evidence of horizontal pleiotropy.

## 4. Discussion

In this study, bidirectional MR analysis was employed to investigate the association between CIPs and gout, with the aim of providing genetic evidence for a potential causal relationship between these biomarkers and gout risk. Our findings suggest that elevated levels of FGF21, MMP-1, G-CSF, and IFN-γ are positively correlated with the onset of gout, as indicated by the forward MR analysis. Furthermore, the reverse MR analysis suggests that elevated levels of CXCL1, IL-1Ra, and TNF-α may be a consequence of gout, with these associations remaining robust after sensitivity analysis. Additionally, a meta-analysis of 23 pairs of CIPs from 2 independent datasets identified an association between increased gout risk and elevated levels of CXCL9 (MIG). Notably, this inflammatory marker did not show a significant effect in the individual MR analyses, likely due to insufficient statistical power in the smaller datasets. However, the meta-analysis, which benefited from a larger sample size and greater statistical power, revealed a significant effect. Despite this, the meta-analysis in this study was based on a relatively small sample size, and as such, it may be susceptible to residual confounding and selection bias. Furthermore, funnel plots could not reliably assess publication bias, leaving open the possibility of false-positive results. Therefore, larger-scale, independent MR studies and randomized controlled trials are warranted in the future to further validate and clarify these findings.^[7,33,34]^

CIPs contribute to the inflammatory process in gout, primarily through the activation of Toll-like receptors (TLRs) and the NOD-like receptor pyrin domain containing 3 (NLRP3) inflammasome by MSU crystals, which leads to the release of pro-inflammatory mediators. Initially, macrophages phagocytose MSU crystals and activate NADPH oxidase, resulting in the production of reactive oxygen species (ROS), which in turn trigger innate immune responses via Toll-like receptor 2 (TLR2) and Toll-like receptor 4 (TLR4).^[35]^ Phagocytosis of MSU crystals further activates the NLRP3 inflammasome, which leads to the activation of caspase-1 and the subsequent generation of IL-1β. This cascade promotes the release of additional inflammatory mediators, including TNF-α, IL-6, and IL-8. These factors interact synergistically, facilitating the recruitment and activation of neutrophils, thereby exacerbating inflammation and ultimately resulting in the clinical manifestations of acute gout attacks, such as severe joint pain, swelling, and erythema.^[36,37]^ The association of IL-1β, TNF-α IL-6, and IL-8 with gout has been well-established. However, the roles of other CIPs in the pathogenesis of gout remain to be fully elucidated, and effective treatments for acute gouty arthritis remain insufficient.^[38,39]^ Consequently, investigating the involvement of CIPs in the inflammatory processes underlying gout is critical for the development of targeted interventions, potentially leading to novel therapeutic strategies for managing gout.

The Fibroblast Growth Factor (FGF) family comprises structurally homologous polypeptides that orchestrate fundamental biological processes including cellular proliferation, differentiation, and metabolic homeostasis.^[40,41]^ Of pathophysiological relevance, FGF21 has emerged as a pleiotropic metabolic modulator exhibiting context-dependent duality in gout pathogenesis. This endocrine effector, predominantly synthesized in hepatocytes and adipocytes, governs systemic glucose homeostasis, lipolysis, and insulin sensitivity through peroxisome proliferator-activated receptor α (PPARα)-dependent mechanisms.^[42,43]^ Genome-wide association studies implicate FGF21 as a candidate causal mediator in gout susceptibility, with a lead variant identified among 26 million participants.^[44]^ Studies have also shown that FGF21 is involved in the regulation of uric acid metabolism, with its levels increasing in hyperuricemia.^[45,46]^ Additional studies have confirmed that FGF21 signaling is associated with reduced uric acid levels and a decreased risk of gout, highlighting its potential as a novel therapeutic approach for the treatment of gout.^[47]^ Paradoxically, while preclinical models demonstrate FGF21’s capacity to attenuate hyperuricemia via dual suppression of NF-κB/NLRP3 inflammasome activation and TGF-1β/Smad3-driven renal fibrosis,^[48–53]^ our bidirectional MR analysis reveals a positive causal association between elevated circulating FGF21 levels and incident gout risk. This discrepancy could reflect tissue-specific effects: experimental evidence indicates that FGF21 enhances mitochondrial function in hepatocytes but may exacerbate oxidative stress in macrophages during inflammatory responses to MSU crystals, potentially through redox-sensitive NLRP3 activation. Clinically, although phase II trials of FGF21 analogs (e.g., efruxifermin) demonstrate significant urate-lowering efficacy in metabolic syndrome cohorts,^[46]^ our genetic evidence advocates for stratified intervention strategies. Prospective pharmacovigilance studies should evaluate whether concomitant administration of NLRP3 inhibitors (e.g., colchicine) or IL-1β-neutralizing biologics could mitigate FGF21-associated gout flare risk in predisposed individuals.

MMP-1, a zinc-dependent collagenase, emerges as a pivotal mediator in gout pathogenesis through its dual capacity to degrade articular extracellular matrix (ECM) and amplify sterile inflammation.^[54]^ Mechanistically, MSU crystals synergistically activate fibroblast-like synoviocytes (FLS) via TLR4/NF-κB signaling, triggering MMP-1 overexpression that is further potentiated by IL-17-mediated stabilization of MMP-1 mRNA.^[55–57]^ This protease cascade facilitates synovial invasion by promoting monocyte-to-macrophage differentiation through keratin-related protein KPT16 upregulation, while collagenolytic cleavage releases proline-glycine-proline (PGP) fragments that chemoattract neutrophils, thereby establishing a self-perpetuating cycle of joint destruction.^[56,58]^ Clinically, our MR findings align with cohort studies demonstrating that synovial fluid MMP-1 levels correlate with ultrasound-defined bone erosion severity in gout,^[59]^ positioning it as both a biomarker for early erosive changes and a therapeutic target. Translationally, selective MMP-1 inhibitors (e.g., AZD1236), currently in phase II trials for osteoarthritis, warrant evaluation in refractory gout patients with radiographically confirmed erosions, particularly when combined with IL-1β blockade to concurrently mitigate inflammasome activation and ECM degradation – a precision strategy that may halt structural progression in this clinically aggressive subtype.

G-CSF, a 25 kDa glycoprotein encoded by the CSF3 gene, emerges as a pivotal orchestrator of neutrophilic inflammation in gout pathogenesis. Beyond its canonical role in granulopoiesis via Janus Kinase 2/Signal Transducer and Activator of Transcription 3 (JAK2/STAT3) signaling,^[60]^ single-cell RNA sequencing reveals G-CSF’s metabolic reprogramming of neutrophils – enhancing PFKFB3-driven glycolysis to fuel neutrophil extracellular trap (NET) formation in response to MSU crystals.^[61]^ This feedforward inflammatory loop is amplified by MSU-induced histone H3K27 acetylation at the CSF3 promoter in synovial macrophages, which synergizes with IL-1β to upregulate G-CSF production by 12-fold compared to controls.^[62]^Clinically, emerging evidence suggests that elevated levels of certain inflammation-associated biomarkers may correlate with therapeutic response in acute gout flares, and their detection may inform the selection of clinical intervention strategies. In the context of inflammatory pathway modulation, targeted therapeutic agents affecting key cytokine-mediated cascades demonstrate potential in regulating neutrophil activity. Furthermore, combination strategies targeting multiple inflammatory pathways are hypothesized to ameliorate pathological progression in complex cases. It should be emphasized that these mechanistic hypotheses and clinical observations require further validation through rigorously designed large-scale studies.

IFN-γ, a pleiotropic cytokine secreted by activated T cells and NK cells, drives gout pathogenesis through dual metabolic-inflammatory mechanisms.^[63–65]^ First, IFN-γ induces hepatic xanthine oxidoreductase (XOR) expression via STAT1-mediated transcriptional activation, accelerating purine catabolism and uric acid overproduction.^[63,64]^ Second, it synergizes with MSU crystals to potentiate NLRP3 inflammasome assembly in macrophages, amplifying IL-1β/IL-18-dependent synovitis.^[65]^ Clinically, serum IFN-γ levels correlate with hyperuricemia severity and predict corticosteroid resistance in acute flares, positioning it as a theranostic biomarker.^[65]^ Notably, while circulating mucosal-associated invariant T (MAIT) cells are depleted in gout patients, residual populations maintain robust IFN-γ/IL-17 production, perpetuating tissue inflammation.^[66]^ Therapeutic breakthroughs emerge from targeting this axis: Sirt1 agonists suppress IFN-γ-induced CXCL1/MCP-1 chemokine storms while normalizing uricemia.^[67]^ and JAK inhibitors (e.g., baricitinib) blunt IFN-γ signaling through STAT3 blockade.^[68]^ In the present study, we also found that elevated levels of IFN-γ may increase the risk of gout, Our findings redefine gout as a metabolic-immunological interface disorder, advocating combined urate-lowering and pathway-specific immunomodulation to address refractory cases.

CXCL1 orchestrates neutrophil-dominated inflammation in gout through feedforward loops with NLRP3 inflammasome activation. Mechanistically, IL-1β-induced CXCL1 secretion recruits neutrophils via CXCR2 binding, while deposited MSU crystals synergistically enhance NF-κB-mediated CXCL1 transcription and NLRP3/ASC/pro-caspase-1 assembly, creating self-perpetuating inflammation.^[69,70]^ Clinically, synovial CXCL1 levels correlate with acute flare severity and predict colchicine resistance, positioning it as a theranostic biomarker for stratified therapy.^[71]^ Quratein D, a CXCL1-neutralizing biologic, suppressing IL-1β/CXCL1 cross-activation in synovium.^[72]^ Lifestyle intervention via moderate-intensity exercise downregulates CXCL1 expression through TLR2/PPARγ-mediated epigenetic silencing of the CXCL1 promoter.^[73]^ Our findings validate CXCL1’s pathogenic centrality and propose combinatorial blockade of Glutamic acid-Leucine-Arginine(ELR)⁺ chemokine networks (CXCL1/CXCL8-CXCR1/2) as a precision strategy for refractory gout.^[74,75]^

IL-1Ra serves as a critical endogenous regulator of gout inflammation through competitive IL-1 receptor blockade. A multi-cohort analysis reveals serum IL-1Ra levels correlate with both hyperuricemia severity and acute-phase C-reactive protein,^[76]^ suggesting its dual role as a disease activity biomarker and compensatory anti-inflammatory responder. Our study also corroborates these findings, demonstrating that gouty arthritis can lead to elevated levels of IL-1Ra. Experimental models further demonstrate 3.8-fold IL-1Ra elevation in rabbit synovial fluid during MSU crystal-induced acute flares,^[77]^ mechanistically explained by NLRP3 inflammasome activation triggering negative feedback via PGE2/EP4-mediated IL-1Ra production. Crucially, synovial IL-1Ra concentrations inversely associate with radiographic progression,^[57]^ supporting combination therapy with recombinant IL-1Ra (e.g., anakinra) and xanthine oxidase inhibitors to simultaneously counterbalance IL-1β excess and urate overproduction. Our findings validate IL-1Ra as a dynamic therapeutic target, with phase-resolved monitoring (acute vs intercritical) guiding personalized IL-1 blockade strategies.

TNF-α orchestrates gout pathogenesis through dual-phase immunometabolic crosstalk. During the initiation phase, MSU crystals activate synovial macrophages via TLR2/4-MyD88 signaling, triggering NF-κB-dependent transcription of both membrane-bound (mTNF-α) and soluble TNF-α (sTNF-α).^[78,79]^ Clinically, serum TNF-α levels correlate with acute flare intensity and predict tophus progression,^[80,81]^ while the TNF-α -863C/A polymorphism increases gout susceptibility 2.3-fold.^[79]^ One such agent is Infliximab, a recombinant monoclonal antibody that specifically binds to and neutralizes TNF-α, thereby blocking its pro-inflammatory effects and providing effective treatment for gout.^[82]^ Currently, therapies targeting IL-1β and TNF-α are promising treatment options, though further clinical research is needed. This study also confirms the positive correlation between TNF-α and gout, offering valuable insights for future drug development and clinical research.

This study offers several significant advantages. Firstly, it represents the first bidirectional MR investigation into the causal relationship between CIPs and gout. In comparison to traditional observational studies, MR is less susceptible to bias arising from confounding variables, environmental factors, and reverse causality, thus providing more robust and reliable results. Secondly, this study is currently the largest to analyze the causal associations between CIPs and gout, incorporating a greater number of CIPs than previous research. Thirdly, we utilized a variety of MR methods, including IVW, weighted median, MR-Egger, and weighted mode, to ensure the robustness and reliability of the findings. While our MR analysis provides novel insights into the causal relationships between CIPs and gout, several limitations warrant consideration. First, although robust genetic instruments (*F*-statistics > 10) were employed, the statistical power to detect small effect sizes remained limited for certain CIPs due to moderate exposure GWAS sample sizes, increasing the likelihood of false-negative results. Second, our analysis assumes linearity in exposure-outcome relationships and cannot entirely exclude residual pleiotropy, though sensitivity analyses (MR-Egger, weighted median) supported result robustness. Additionally, the predominantly Finnish and European ancestry of the study populations may introduce genetic heterogeneity (e.g., historical population bottlenecks, geographical isolation, genetic drift), as evidenced by discrepancies in causal estimates for specific CIPs (e.g., IFN-γ and CXCL1) between the GWAS Catalog and IEU OpenGWAS databases. Notably, after Bonferroni correction for multiple testing, several associations lost statistical significance, potentially reflecting stringent correction-induced type II errors. Furthermore, inconsistent causal links were observed for established inflammatory mediators (e.g., TNF-α, IL-1β, IL-6, IL-8), possibly attributable to limited ancestral diversity or insufficient instrument strength in MR models. To address these limitations and advance translational applications, future studies should prioritize trans-ethnic GWAS meta-analyses to enhance ancestral diversity and statistical power, integrate protein quantitative trait loci (pQTLs) to refine causal inference precision, conduct randomized controlled trials targeting prioritized CIPs (e.g., IL-1β, TNF-α inhibitors), and implement larger-scale MR studies incorporating longitudinal imaging biomarkers (e.g., dual-energy CT urate volume) to resolve inconsistencies and elucidate temporal dynamics.

## 5. Conclusions

In summary, this bidirectional MR study provides more robust evidence than previous observational studies, demonstrating the association between several cytokines and gout. Specifically, we identified multiple CIPs, including FGF21, MMP-1, G-CSF, IFN-γ, CXCL1, IL-1Ra, and TNF-α, which may serve as potential molecular therapeutic targets for gout. While some of these results warrant further validation, this study has successfully pinpointed candidate cytokines that could facilitate future investigations into the pathogenesis of gout. These findings offer a new theoretical foundation for advancing research and treatment strategies for gout. Moreover, future drug development targeting key cytokine functions could lead to novel therapeutic approaches for both the prevention and treatment of gout, with significant implications for the clinical management of the disease.

## Acknowledgments

We extend our gratitude to all the investigators and participants who contributed to the genetic association summary data used in this study

## Author contributions

**Conceptualization:** Xiaochao Xie, Fang Wang.

**Data curation:** Xiaochao Xie, Yanjie Song.

**Formal analysis:** Xiaochao Xie.

**Funding acquisition:** Xiaochao Xie.

**Investigation:** Xiaochao Xie, Yanjie Song.

**Methodology:** Xiaochao Xie.

**Project administration:** Xiaochao Xie.

**Resources:** Xiaochao Xie, Hui Zhao, Nan Chu.

**Software:** Xiaochao Xie, Wenwen Chen.

**Supervision:** Fang Wang.

**Validation:** Xiaochao Xie, Wenwen Chen.

**Visualization:** Xiaochao Xie, Yanjie Song.

**Writing – original draft:** Xiaochao Xie.

**Writing – review & editing:** Xiaochao Xie, Wenwen Chen, Fang Wang.

## Supplementary Material
