# Supplementary material for: Association between circulating inflammatory proteins and gout: A Mendelian randomization study
Source: Medicine (Baltimore). 2025 May 16;104(20):e42379. doi: 10.1097/MD.0000000000042379 (PMC12091660; doi:10.1097/MD.0000000000042379)
Supplement: Supplementary file 2 [file medi-104-e42379-s002.pdf]

Supplementary Figure 1 Forest plots of forward MR between five CIPs and gout.

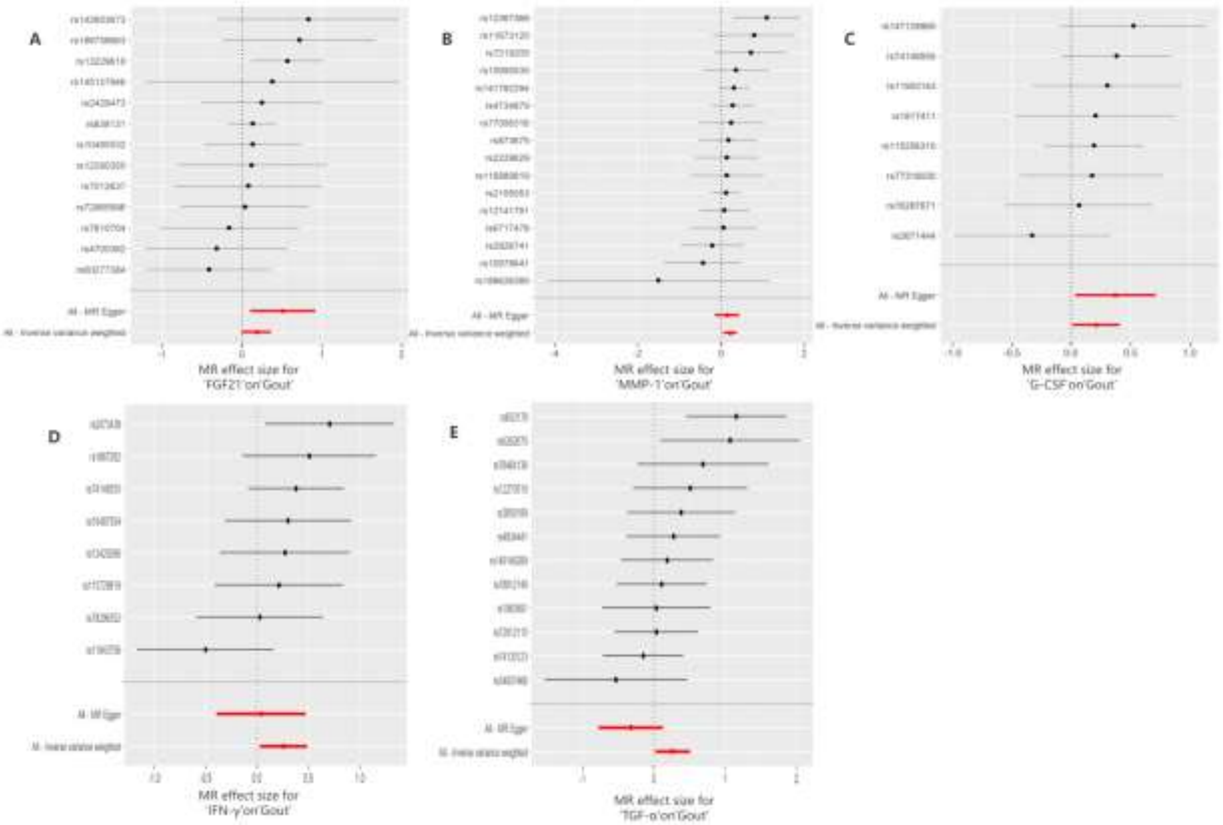

Abbreviations:**(A)** FGF21, Fibroblast Growth Factor 21; **(B)** MMP-1, Matrix metalloproteinase-1; **(C)** G-CSF, Granulocyte Colony-Stimulating Factor; **(D)** IFN-γ, Interferon-goutmma; **(E)**TGF-α, Transforming Growth Factor Alpha; CIPs,Circulating Inflammatory Proteins; CIPs, Circulating Inflammatory Proteins.

Supplementary Figure 2 Leave-one-out sensitivity analyses of forward MR between five CIPs and gout.

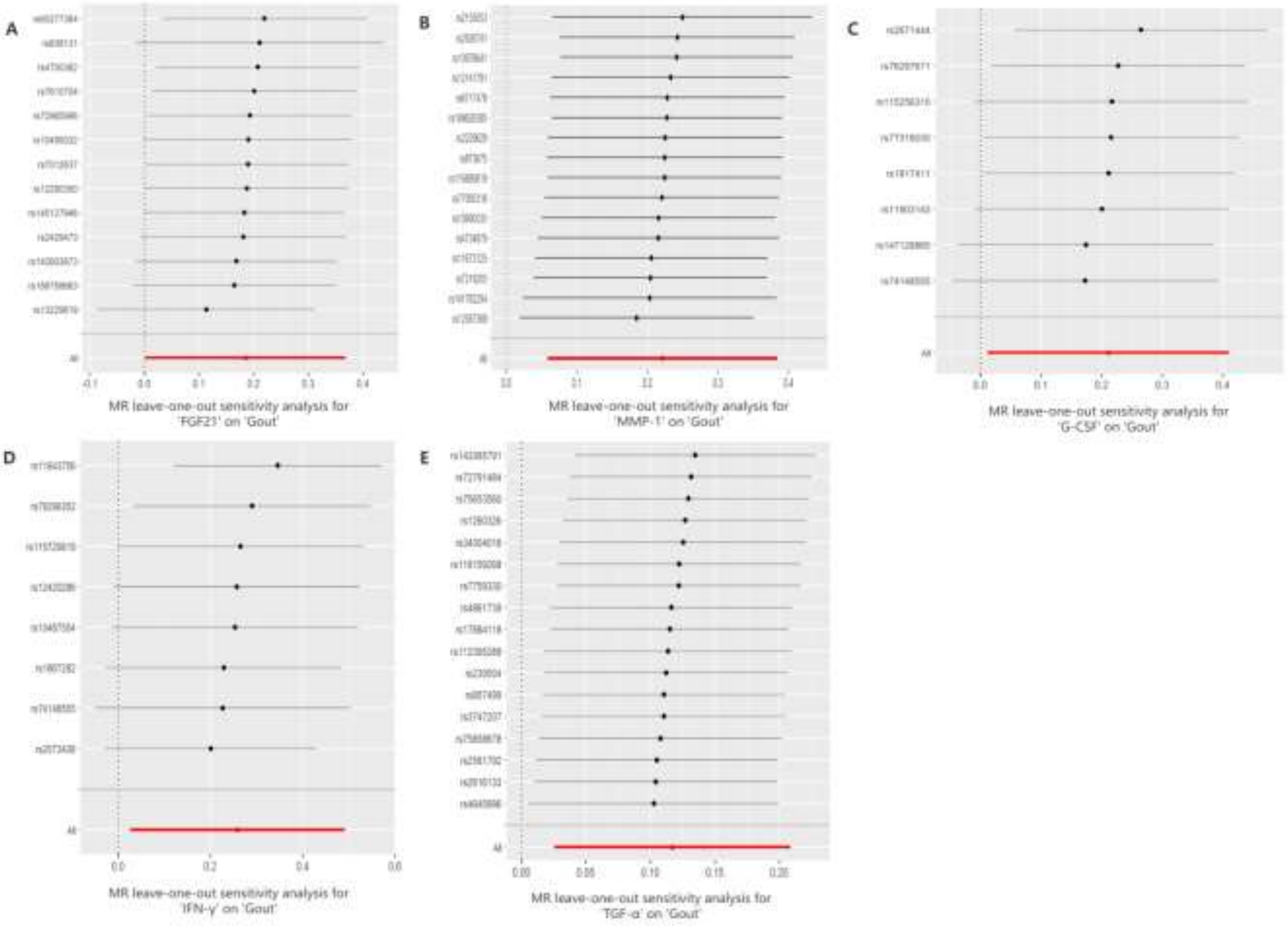

Abbreviations: **(A)** FGF21,Fibroblast Growth Factor 21; **(B)** MMP-1,Matrix metalloproteinase-1; **(C)** G-CSF,Granulocyte Colony-Stimulating Factor; **(D)** IFN- γ ,Interferon-goutmma; **(E)**TGF-α,Transforming Growth Factor Alpha; CIPs,Circulating Inflammatory Proteins

Supplementary Figure 3 The forest plot of 132 CIPs in the forward MR obtained through the inverse variance weighted method, with CIPs as exposure and gout as outcome.

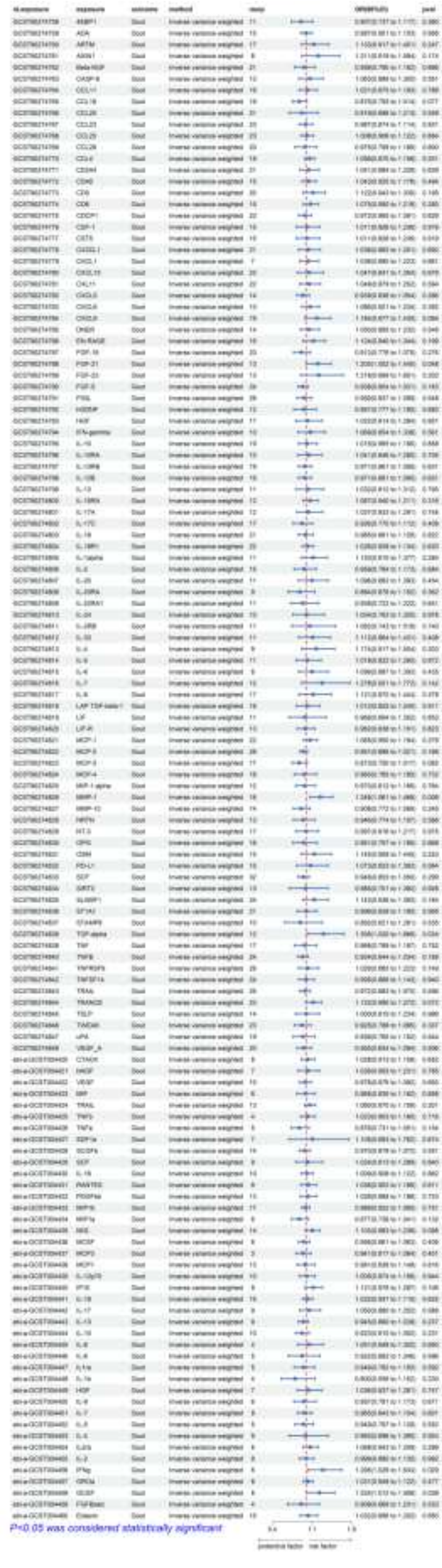
P<0.05 was considered statistically significant

Abbreviations: nsnp, the number of single-nucleotide polymorphisms used in the analysis; OR, Odds Ratio; CIPs, Circulating Inflammatory Proteins; BDNF, brain-derived neurotrophic factor; bNGF, beta-nerve growth factor; CNTF, ciliary neurotrophic factor; CTACK, cutaneous T-cell attracting chemokine; FGFBasic, fibroblast growth factor basic; GCSF, granulocyte colony-stimulating factor; GROa, growth-regulated-alpha; HGF, hepatocyte growth factor; IFNb, interferon-beta; IFNg, interferon-goutmma; IGF1, insulin-like growth factor 1; IL, interleukin; IP10, interferon goutmma-induced protein 10; LIF, leukemia inhibitory factor; MCP1, monocyte chemoattractant protein-1; MCP3, monocyte-specific chemokine 3; MCSF1, Macrophage colony-stimulating factor 1; MIF, macrophage migration inhibitory factor; MIG, monokine induced by interferon goutmma; MIP1a, macrophage inflammatory protein-1a; MIP1b, macrophage inflammatory protein-1b; PDGFB, platelet-derived growth factor subunit A; PDGFB, platelet-derived growth factor subunit B; PDGFbb, platelet-derived growth factor BB; RANTES, regulated upon activation normal T cell expressed and presumably secreted; SCF, stem cell factor;SCGFb, stem cell growth factor beta; SDF1, stromal cell-derived factor-1; TNFα, tumor necrosis factor alpha;TNFb, tumor necrosis factor beta; TRAIL, TNF-related apoptosis inducing ligoutnd; VEGF, vascular endothelial growth factor; VEGFD, vascular endothelial growth factor D; 4EBP1,Eukaryotic translation initiation factor 4E-binding protein 1 levels; ADA,Adenosine Deaminase levels; ARTM, Artemin levels; AXIN1, Axin-1 levels; Beta-NGF, beta-nerve growth factor levels; CASP-8, Caspase 8 levels; CCL11, Eotaxin levels; CCL19, C-C motif chemokine 19 levels; CCL20,C-C motif chemokine 20 levels; CCL23,C-C motif chemokine 23 levels; CCL25,C-C motif chemokine 25 levels; CCL28,C-C motif chemokine 28 levels; CCL4,C-C motif chemokine 4 levels; CD244,Natural killer cell receptor 2B4 levels; CD40, CD40L receptor levels; CD5,T-cell surface glycoprotein CD5 levels; CD6,T-cell surface glycoprotein CD6 isoform levels; CDCP1, CUB domain-containing protein 1 levels; CSF-1, Macrophage colony-stimulating factor 1 levels; CST5, Cystatin D levels; CX3CL1,Fractalkine levels; CXCL1, C-X-C motif chemokine 1 levels; CXCL10, C-X-C motif chemokine 10 levels; CXL11, C-X-C motif chemokine 11 levels; CXCL5, C-X-C motif chemokine 5 levels; CXCL6, C-X-C motif chemokine 6 levels; CXCL9, C-X-C motif chemokine 9 levels; DNER, Delta and Notch-like epidermal growth factor-related receptor levels; EN-RAGE,Protein S100-A12 levels; FGF-19, Fibroblast growth factor 19 levels; FGF-21, Fibroblast growth factor 21 levels; FGF-23, Fibroblast growth factor 23 levels; FGF-5, Fibroblast growth factor 5 levels; FIt3L, Fms-related tyrosine kinase 3 ligoutnd levels; hGDNF, Glial cell line-derived neurotrophic factor levels; HGF, Hepatocyte growth factor levels; IFN-goutmma, Interferon goutmma levels; IL-10, Interleukin-10 levels; IL-10RA, Interleukin-10 receptor subunit alpha levels; IL-10RB, Interleukin-10 receptor subunit beta levels; IL-12B, Interleukin-12 subunit beta levels; IL-13, Interleukin-13 levels; IL-15RA, Interleukin-15 receptor subunit alpha levels; IL-17A, Interleukin-17A levels; IL-17C, Interleukin-17C levels; IL-18, Interleukin-18 levels; IL-18R1, interleukin-18 receptor 1 levels; IL-1alpha, Interleukin-1-alpha levels; IL-2,Interleukin-2 levels;IL-20, Interleukin-20 levels; IL-20RA, Interleukin-20 receptor subunit alpha levels; IL-22RA1,Interleukin-22 receptor subunit alpha-1 levels; IL-24, Interleukin-24 levels; IL-2RB, Interleukin-2 receptor subunit beta levels; IL-33,Interleukin-33 levels; IL-4, Interleukin-4 levels; IL-5, Interleukin-5 levels; IL-6, Interleukin-6 levels; IL-7, Interleukin-7 levels; IL-8, Interleukin-8 levels; LAP TGF-beta-1, Latency-associated peptide transforming growth factor beta 1 levels; LIF, Leukemia inhibitory factor levels; LIF-R, Leukemia inhibitory factor receptor levels; MCP-1,Monocyte chemoattractant protein-1 levels; MCP-2,Monocyte chemoattractant protein 2 levels; MCP-3, Monocyte chemoattractant protein-3 levels; MCP-4,Monocyte chemoattractant protein-4 levels; MIP-1 alpha, Macrophage inflammatory protein 1a levels; MMP-1, Matrix metalloproteinase-1 levels; MMP-10, Matrix metalloproteinase-10 levels; NRTN,Neurturin levels; NT-3, Neurotrophin-3 levels; OPG,Osteoprotegerin levels; OSM, Oncostatin-M levels; PD-L1,Programmed cell death 1 ligoutnd 1 levels; SCF, Stem cell factor levels; SIRT2, SIR2-like protein 2 levels; SLAMF1, Signaling lymphocytic activation molecule levels; ST1A1, Sulfotransferase 1A1 levels;

STAMP8, STAM binding protein levels; TGF-alpha, Transforming growth factor-alpha levels; TNF, Tumor necrosis factor levels; TNFB, TNF-beta levels; TNFRSF9, Tumor necrosis factor receptor superfamily member 9 levels; TNFSF14, Tumor necrosis factor ligoutnd superfamily member 14 levels; TRAIL, TNF-related apoptosis-inducing ligoutnd levels; TRANCE,TNF-related activation-induced cytokine levels; TSLP,Thymic stromal lymphopoietin levels; TWEAK, Tumor necrosis factor ligoutnd superfamily member 12 levels; uPA,Urokinase-type plasminogen activator levels; VEGF_A,Vascular endothelial growth factor A levels; CIPs,Circulating Inflammatory Proteins.

Supplementary Figure 4 Forest plots of reverse MR analyses between gout and three CIPs.

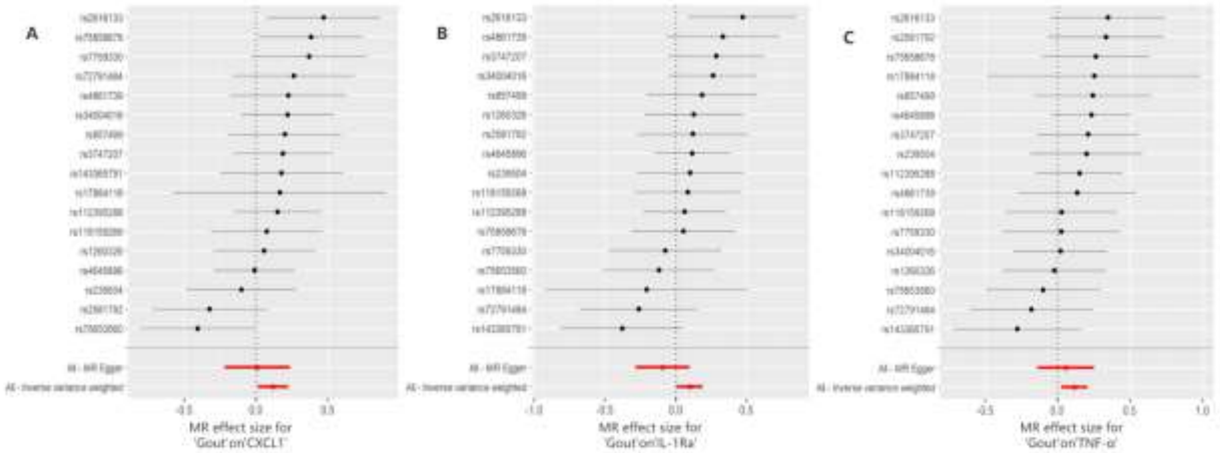

Abbreviations:(A) CXCL1,CXC motif chemokine ligoutnd 1;**(B)** IL-1Ra,Interleukin 1 Receptor Antagonist;**(C)** TNF-α ,Tumor Necrosis Factor-alpha;CIPs,Circulating Inflammatory Proteins

Supplementary Figure 5 "Leave-one-out" sensitivity analyses of reverse MR analyses between gout and three CIPs.

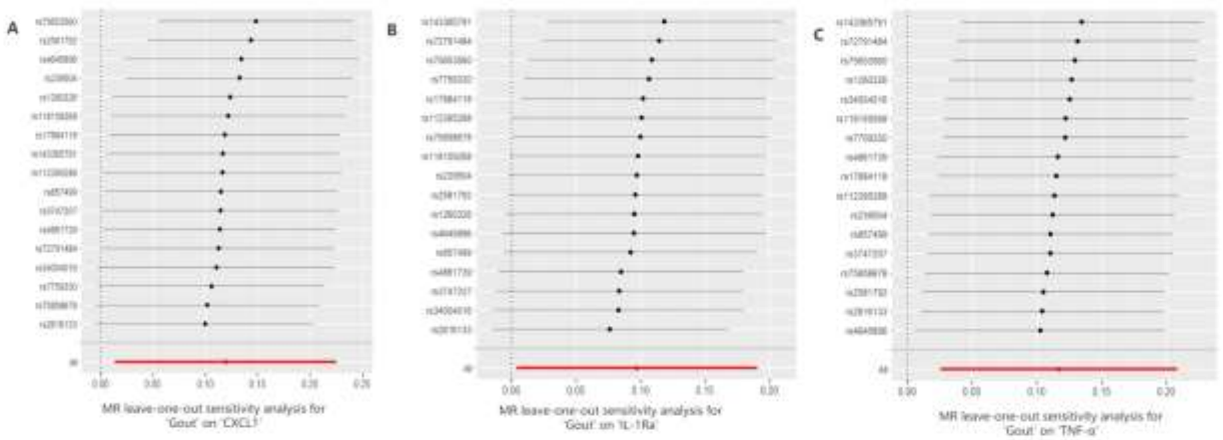

Abbreviations:(A) CXCL1, CXC motif chemokine ligoutnd 1; **(B)** IL-1Ra,Interleukin 1 Receptor Antagonist; **(C)** TNF-α , Tumor Necrosis Factor-alpha; CIPs, Circulating Inflammatory Proteins.

Supplementary Figure 6 The forest plot of 132 CIPs in the reverse MR analysis obtained through the inverse variance weighted method,with gout as exposure and CIPs as outcome.

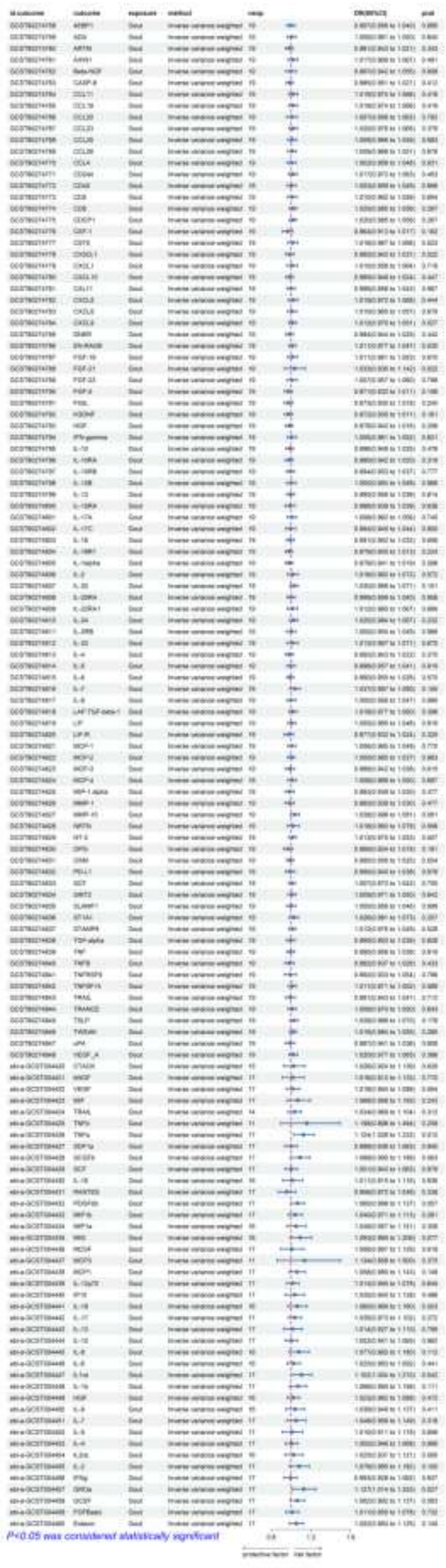
P<0.05 was considered statistically significant

Abbreviations: nsnp, the number of single-nucleotide polymorphisms used in the analysis; OR, Odds Ratio gout, gouty arthritis; CIPs, Circulating Inflammatory Proteins; BDNF, brain-derived neurotrophic factor; bNGF, beta-nerve growth factor; CNTF, ciliary neurotrophic factor; CTACK, cutaneous T-cell attracting chemokine; FGFBasic, fibroblast growth factor basic; GCSF, granulocyte colony-stimulating factor; GROa, growth-regulated-alpha; HGF, hepatocyte growth factor; IFNb, interferon-beta; IFNg, interferon-goutmma; IGF1, insulin-like growth factor 1; IL, interleukin; IP10, interferon goutmma-induced protein 10; LIF, leukemia inhibitory factor; MCP1, monocyte chemoattractant protein-1; MCP3, monocyte-specific chemokine 3; MCSF1, Macrophage colony-stimulating factor 1; MIF, macrophage migration inhibitory factor; MIG, monokine induced by interferon goutmma; MIP1a, macrophage inflammatory protein-1a; MIP1b, macrophage inflammatory protein-1b; PDGFB, platelet-derived growth factor subunit A; PDGFB, platelet-derived growth factor subunit B; PDGFbb, platelet-derived growth factor BB; RANTES, regulated upon activation normal T cell expressed and presumably secreted; SCF, stem cell factor;SCGFb, stem cell growth factor beta; SDF1, stromal cell-derived factor-1; TNFα, tumor necrosis factor alpha;TNFb, tumor necrosis factor beta; TRAIL, TNF-related apoptosis inducing ligoutnd; VEGF, vascular endothelial growth factor; VEGFD, vascular endothelial growth factor D; 4EBP1,Eukaryotic translation initiation factor 4E-binding protein 1 levels; ADA,Adenosine Deaminase levels; ARTM, Artemin levels; AXIN1, Axin-1 levels; Beta-NGF, beta-nerve growth factor levels; CASP-8, Caspase 8 levels; CCL11, Eotaxin levels; CCL19, C-C motif chemokine 19 levels; CCL20,C-C motif chemokine 20 levels; CCL23,C-C motif chemokine 23 levels; CCL25,C-C motif chemokine 25 levels; CCL28,C-C motif chemokine 28 levels; CCL4,C-C motif chemokine 4 levels; CD244,Natural killer cell receptor 2B4 levels; CD40, CD40L receptor levels; CD5,T-cell surface glycoprotein CD5 levels; CD6,T-cell surface glycoprotein CD6 isoform levels; CDCP1, CUB domain-containing protein 1 levels; CSF-1, Macrophage colony-stimulating factor 1 levels; CST5, Cystatin D levels; CX3CL1,Fractalkine levels; CXCL1, C-X-C motif chemokine 1 levels; CXCL10, C-X-C motif chemokine 10 levels; CXL11, C-X-C motif chemokine 11 levels; CXCL5, C-X-C motif chemokine 5 levels; CXCL6, C-X-C motif chemokine 6 levels; CXCL9, C-X-C motif chemokine 9 levels; DNER, Delta and Notch-like epidermal growth factor-related receptor levels; EN-RAGE,Protein S100-A12 levels; FGF-19, Fibroblast growth factor 19 levels; FGF-21, Fibroblast growth factor 21 levels; FGF-23, Fibroblast growth factor 23 levels; FGF-5, Fibroblast growth factor 5 levels; FIt3L, Fms-related tyrosine kinase 3 ligoutnd levels; hGDNF, Glial cell line-derived neurotrophic factor levels; HGF, Hepatocyte growth factor levels; IFN-goutmma, Interferon goutmma levels; IL-10, Interleukin-10 levels; IL-10RA, Interleukin-10 receptor subunit alpha levels; IL-10RB, Interleukin-10 receptor subunit beta levels; IL-12B, Interleukin-12 subunit beta levels; IL-13, Interleukin-13 levels; IL-15RA, Interleukin-15 receptor subunit alpha levels; IL-17A, Interleukin-17A levels; IL-17C, Interleukin-17C levels; IL-18, Interleukin-18 levels; IL-18R1, interleukin-18 receptor 1 levels; IL-1alpha, Interleukin-1-alpha levels; IL-2,Interleukin-2 levels;IL-20, Interleukin-20 levels; IL-20RA, Interleukin-20 receptor subunit alpha levels; IL-22RA1,Interleukin-22 receptor subunit alpha-1 levels; IL-24, Interleukin-24 levels; IL-2RB, Interleukin-2 receptor subunit beta levels; IL-33,Interleukin-33 levels; IL-4, Interleukin-4 levels; IL-5, Interleukin-5 levels; IL-6, Interleukin-6 levels; IL-7, Interleukin-7 levels; IL-8, Interleukin-8 levels; LAP TGF-beta-1, Latency-associated peptide transforming growth factor beta 1 levels; LIF, Leukemia inhibitory factor levels; LIF-R, Leukemia inhibitory factor receptor levels; MCP-1,Monocyte chemoattractant protein-1 levels; MCP-2,Monocyte chemoattractant protein 2 levels; MCP-3, Monocyte chemoattractant protein-3 levels; MCP-4,Monocyte chemoattractant protein-4 levels; MIP-1 alpha, Macrophage inflammatory protein 1a levels; MMP-1, Matrix metalloproteinase-1 levels; MMP-10, Matrix metalloproteinase-10 levels; NRTN,Neurturin levels; NT-3, Neurotrophin-3 levels; OPG,Osteoprotegerin levels; OSM, Oncostatin-M levels; PD-L1,Programmed cell death 1 ligoutnd 1 levels; SCF, Stem cell factor levels; SIRT2, SIR2-like protein 2 levels; SLAMF1, Signaling lymphocytic activation molecule levels; ST1A1,

Sulfotransferase 1A1 levels; STAMP8, STAM binding protein levels; TGF-alpha, Transforming growth factor-alpha levels; TNF, Tumor necrosis factor levels; TNFB, TNF-beta levels; TNFRSF9, Tumor necrosis factor receptor superfamily member 9 levels; TNFSF14, Tumor necrosis factor ligoutnd superfamily member 14 levels; TRAIL, TNF-related apoptosis-inducing ligoutnd levels; TRANCE,TNF-related activation-induced cytokine levels; TSLP,Thymic stromal lymphopoietin levels; TWEAK, Tumor necrosis factor ligoutnd superfamily member 12 levels; uPA,Urokinase-type plasminogen activator levels; VEGF_A,Vascular endothelial growth factor A levels; CIPs,Circulating Inflammatory Proteins.

Supplementary Figure 7 Results of statistical power calculations for the positive CIPs.

**CIP**	**N_outcome**	**R^2**	β	α	**Power**
FGF21	150797	0.036	0.185	0.05	100%
MMP1	150797	0.102	0.221	0.05	100%
GCSF	150797	0.032	0.211	0.05	100%
IFN-γ	150797	0.030	0.259	0.05	100%
CXCL1	14824	0.344	0.119	0.05	100%
IL1Ra	14824	0.344	0.097	0.05	100%
TNF-α	14824	0.344	0.117	0.05	100%

Abbreviations:**(A)** FGF21, Fibroblast Growth Factor 21; **(B)** MMP-1, Matrix metalloproteinase-1; **(C)** G-CSF, Granulocyte Colony-Stimulating Factor; **(D)** IFN-γ, Interferon-goutmma; **(E)** CXCL1, CXC motif chemokine ligoutnd 1; **(F)** IL-1Ra,Interleukin 1 Receptor Antagonist; **(G)** TNF-α, Tumor Necrosis Factor-alpha; CIPs, Circulating Inflammatory Proteins.
